# Exploring the spatial-temporal distribution and evolution of population aging and social-economic indicators in China

**DOI:** 10.1186/s12889-021-11032-z

**Published:** 2021-05-22

**Authors:** Wang Man, Shaobin Wang, Hao Yang

**Affiliations:** 1 Department of Spatial Information Science and Engineering, Xiamen University of Technology, Xiamen, 361024 China; 2 Institute of Geographic Sciences and Natural Resources Research, Chinese Academy of Sciences, A11 Datun Road, Anwai, Beijing, 100101 China; 3 Beijing Academy of Social Sciences, Beijing, 100101 China; 4 School of Economics, Peking University, Beijing, 100871 China

**Keywords:** Spatial-temporal patterns, Population aging indicators, Per capita GRP, Gravity centers, Social-economic factors

## Abstract

**Background:**

China is one of the world’s fastest-aging countries. Population aging and social-economic development show close relations. This study aims to illustrate the spatial-temporal distribution and movement of gravity centers of population aging and social-economic factors and thier spatial interaction across the provinces in China.

**Methods:**

Factors of elderly population rate (EPR), elderly dependency ratio (EDR), per capita gross regional product (GRP_pc_), and urban population rate (UPR) were collected. Distribution patterns were detected by using global spatial autocorrelation, Kernel density estimation, and coefficient of variation. Further, Arc GIS software was used to find the gravity centers and their movement trends yearly from 2002 to 2018. The spatial interaction between the variables was investigated based on bivariate spatial autocorrelation analysis.

**Results:**

The results showed a larger variety of global spatial autocorrelation indexed by Moran’s *I* and stable trends of dispersion degree without obvious convergence in EPR and EDR. Furthermore, the gravity centers of the proportion of EPR and EDR moved northeastward. In contrast, the economic and urbanization factors showed a southwestward movement, which exhibited an reverse trend compared to population aging indicators. Moreover, the movement rates of EPR and EDR (15.12 and 18.75 km/year, respectively) were higher than that of GRP_pc_ (13.79 km/year) and UPR (6.89 km/year) annually during the study period. Further, the bivariate spatial autocorrelation variation is in line with the movement trends of gravity centers which showed a polarization trend of population aging and social-economic factors that the difference between southwest and northeast directions and exhibited a tendency to expand in China.

**Conclusions:**

In sum, our findings revealed the difference in spatio-temporal distribution and variation between population aging and social-economic factors in China. It further indicates that the opposite movements of gravity centers and the change of the BiLISA in space which may result in the increase of the economic burden of the elderly care in northern China. Hence, future development policy should focus on the social-economic growth and distribution of old-aged supporting resources, especially in northern China.

**Supplementary Information:**

The online version contains supplementary material available at 10.1186/s12889-021-11032-z.

## Background

The elderly population is increasing in number and in the share of the total population, which is becoming one of the most severe demographic challenges in the world [1]. Population aging and social-economic development show close relations. For one thing, the potential influence of population aging on the economy and society has been widely discussed and acknowledged [2–6]. For another, national social-economic development and poverty-reduction strategies were considered to be priority actions to meet the challenge of an aging society in the world [7]. Meanwhile, the spatial distribution of the elderly population exhibits an obvious difference at the global level [8, 9] and country-level such as China [10], Japan [11], South Korea [12], and some European countries [13].

In fact, the concept of a center of gravity mainly refers to the point on which the distribution pattern would balance by using weighted points on a weightless plane or sphere [14]. This concept was first applied to probe the population problems in the United States, which provided a concise and accurate method for the population distribution study [15]. Then, this concept has been applied in the study of geographic distributions [16, 17]. As with other geographical phenomena, spatial distribution patterns and processes of population aging also vary over time. Thus, appropriate assessment of population aging and social-economic development should be able to include the features of spatial-temporal variations with the change of gravity centers in geographic space, which can be expected to provide more in-depth implications for the policymaking of the elderly-care and sustainable socio-economic development.

China, with an increased ratio and size of the elderly population, is one of the world’s fastest-aging countries [18]. In 2019, about 11.5% of the total population were above 65 years old in China, and it is expected to reach 16.9% by 2030, estimated by the United Nations [19]. The accelerated population aging process may place a heavy burden on China’s social security and economic systems, thus, maintaining the well-being of an elderly population has become a major concern for policymakers. Extensive studies have been conducted on spatial-temporal distribution and variation of population aging in China [10, 20–25]. Huge spatial differences of the elderly population were found at the national level and provincial levels in China based on these studies. Importantly, spatial effects such as spatial spillovers and spatial dependence were found in geographic data of population aging and social-economic factors [9, 23, 24]. Nevertheless, there have been no studies of the distribution of gravity centers in population aging and how the patterns changed over time in China. Furthermore, the difference in the spatial-temporal distribution and evolution of population aging and social-economic factors in China is obscure as well.

Consequently, in this study, we mainly focused on the evolution of the patterns of population aging indicators and social-economic factors at the provincial level from 2002 to 2018 based on their centers of gravity in China. Moreover, different from previous studies in China, two main population aging indicators were collected and calculated in this study to measure the level of aging which included the elderly population rate and elderly dependency ratio which may show more social-economic meanings than before. In addition, per capita Gross Regional Product and urban population rate were collected as proxy indicators to measure the social-economic development levels. In this paper, the methods of global spatial autocorrelation, kernel density estimation, and coefficient of variation were performed to measure the spatial patterns. Further, evolution patterns analysis was performed by using the method of gravity center detection, and the spatial interaction between variables was investigated based on bivariate spatial correlation analysis. In sum, our findings can reveal the distribution and variation of population aging and social-economic factors and contribute to a better understanding and the implications to the coordinated social-economic development in China that is facing severe challenges from an aging society.

## Data and methods

### Data sources and indicator selection

#### Data source

In the current paper, the demographic data in 2010 were based on the national census of China [26]. Data in 2005 and 2015 were from the 1% Population Sample Survey, and data in other years were from the 1‰ Population Sample Survey of China. These census data are at the provincial level (including province-level autonomous regions and municipalities) in mainland China. The data of the social-economic indicators were from the *China Statistical Yearbooks*. The collected panel data included the provincial administrative units of mainland China with sufficient data and covers the period 2002–2018. Hong Kong, Macau, and Taiwan were not included in this study for the non-available data.

#### Population aging indicators

##### Elderly population rate

The elderly population rate (EPR) refers to the percent of the aged population of the total population, which has been widely applied in the studies of population aging [1, 7, 27]. As the standard threshold for population aging, 60 or 65 years old were used as commonly accepted national retirement ages [28]. In this study, the elderly population is defined as people aged 65 and over based on the current chronological-age structure in China [29]. The ratio of population aged 65 and above (% of the total population) was calculated as one of the indicators to measure the population aging at the provincial level in China.

##### Elderly dependency ratio

The elderly dependency ratio (EDR) is commonly defined as the ratio between the elderly population and the working-age population [30]. This indicator has been widely used as an indicator to monitor the population’s age structures and to provide implications on socioeconomic development [31, 32]. In this paper, in line with UN definitions, the population aged between 15 and 64 are considered as the margins of the working-age population. Thus, the ratio of population aged and above 65 and the working-age population is considered another indicator to measure the population aging level in this study.

#### Social-economic factors

The association between population aging and social-economic development (e.g., GDP and urbanization levels) has been widely conducted at the country level [23, 33, 34]. Therefore, in this study, two main social-economic factors including per capita Gross Regional Product (GRP_pc_) and urban population rate (UPR) were collected at the provincial level in China (Table 1). The UPR was calculated as the proportion (%) of people living in urban areas of the total population.

**Table 1:** Indicator selected in the study (2002–2018)

Variable		Abbreviation	Unit
Population aging indicators	Elderly population rate	EPR	%
	Elderly dependency ratio	EDR	%
Social-economic indicators	Per capita Gross Regional Product	GRP_pc_	RMB yuan
	Urban population rate	UPR	%

### Methodology

#### Distribution pattern detection

##### Global spatial autocorrelation

Global spatial autocorrelation indexed by Moran’s *I* method is a powerful tool to quantitatively provide the indicators varying geographically and to explore the spatial autocorrelation among samples [35]. This method has been widely applied to examine spatial patterns, i.e., clustered, dispersed, or random distributions [36–39]. To explore the spatial autocorrelation of population aging indicators, Moran’s *I* was adopted in this paper, which can be calculated as follows [40]:

1 $$ I=\frac{n\times {\sum}_i^n{\sum}_j^n{W}_{ij}\left({x}_i-\overline{x}\right)\left({x}_j-\overline{x}\right)}{\sum_i^n{\sum}_j^n{W}_{ij}\times {\sum}_i^n{\left({x}_i-\overline{x}\right)}^2} $$

In Eq. (1), *n* represents the number of spatial units (the province in this case) indexed by *i* and *j*; *x* represents the selected variables; $\overline{x}$ is the mean of *x*; the weights *W*_*ij*_ are written in an (*n* × *n*) weight matrix which depicts the relationship between the variable and its surrounding ones. In the current study, the contiguity-based method was conducted for identifying the weight matrix, and the queen criterion as spatial units sharing a common edge or a vertex was selected to determine neighbors. The global spatial autocorrelation was performed by using GeoDa software (version 1.18).

##### Kernel density estimation

Kernel density estimation (KDE) is commonly used to estimate the density function based on a set of observations and random variables from an unknown distribution function. This method does not need any hypothesis for the data distribution. Thus, the general distribution patterns in population aging indicators can be described visually based on the kernel density function curves. In this study, the KDE calculation is presented as follows [41]:

2 $$ f(x)=\frac{1}{nh}{\sum}_{i=1}^tK\left(\frac{x_i-x}{h}\right) $$

In Eq. (2), *n* is the total number of locations, *x*_*i*_ is the value of *i*th population aging indicators, and *h* is the bandwidth. *K* is a kernel density function and the Gaussian kernel was adopted in this paper.

##### Coefficient of variation

The coefficient of variation (COV) is an effective method to measure the dispersion degree of an indicator [42, 43]. The COV is indicated in convergence analysis because it does not depend on the measurement unit and the measure order of the indicators [44]. In this paper, this method is adopted which could be shown as follows:

3 $$ {\sigma}_t=\frac{\sqrt{\frac{1}{n}{\sum}_{i=1}^n{\left({x}_t-{\overline{x}}_t\right)}^2}}{{\overline{x}}_t} $$

In Eq. (3), *t* refers to the year, and *n* denotes the total number of provinces. *x* represents the population aging indicators and $\overline{x}$ is the mean value in year *t*. *σ*_*t*_ represents the COV of the selected indicators. In this paper, the calculation of COV expresses the dispersion degree of the population aging indicators compared to the average levels.

#### Evolution patterns analysis

The center of gravity is a physical concept. Then, it has been widely adopted to investigate spatial distribution, such as population [14], energy consumption [45], carbon emissions [46], and economic growth [47], etc. In this paper, the center of gravity method was used to analyze the spatial evolution of population aging and social-economic indicators. Each provincial-level administrative region is assumed to be located on a homogeneous two-dimension surface without accounting for the altitude dimension, and that the indicators by each region are concentrated in the provincial capital cities. Each provincial capital city then acts as a particle on the surface, with weights determined based on the indicator’s quantity. This simplification is a necessary step because finer-grained data (e.g., at the prefectural and county levels) is not yet available in this study. Then, the spatial positions of the gravity centers were calculated according to a combination of the geographical coordinates of the provincial capital cities and their corresponding weights. The position of the gravity center is expressed in terms of longitude and latitude, which can be calculated as follows:

4 $$ {X}_t=\frac{\sum {M}_{ti}{x}_i}{\sum {M}_{ti}} $$

5 $$ {Y}_t=\frac{\sum {M}_{ti}{y}_i}{\sum {M}_{ti}} $$

In Eq. (4) and (5), *X*_*t*_ and *Y*_*t*_ are the longitude and latitude coordinates of the gravity center in year *t,* respectively. *M*_*ti*_ represents the population aging and social-economic indicators by province *i* in year *t*. (*x*_*i*_, *y*_*i*_) represent the longitude and latitude coordinates of the provincial capital city of province *i*, respectively. Furthermore, the spatial movement distance for the gravity center can be expressed as follows:

6 $$ {D}_{t2-t1}=\sqrt{{\left({X}_{t2}-{X}_{t1}\right)}^2+{\left({Y}_{t2}-{Y}_{t1}\right)}^2} $$

In Eq. (6), *D*_t2-t1_ is the movement distance of the gravity center (km) between the year *t*_2_ and *t*_1_, and *X*_t1_, *X*_t2,_ and *Y*_t1_, *Y*_t2_ are gravity center coordinates for the years *t*_1_ and *t*_2_. The differences in the movement of these gravity centers represent the changes in the balance over time. In this paper, Arc GIS software (version 10.2) was used to calculate the gravity centers in each year and connect each center in turn to define the movement paths followed by these centers annually from 2002 to 2018.

#### Bivariate spatial autocorrelation analysis

Bivariate spatial autocorrelation is considered to be the correlation between one variable and the spatial lag of another variable [48]. The bivariate spatial autocorrelation analysis is commonly indexed by bivariate Moran’s *I*, whereas the original Moran’s *I* statistic measured the degree of linear association of the values of a variable in neighboring regions. The bivariate Moran’s *I* statistic provides an indication of the degree of linear association between one variable and a different variable in neighboring regions. Meanwhile, the bivariate local indicators of spatial association (BiLISA) which closely follows that of its global counterpart was applied in this study to capture the relationship between the value for one variable at a location and the average of the neighboring values for another variable [48]. In this study, bivariate Moran’s *I* between population aging indicators and social-economic factors were calculated and the cluster maps of BiLISA were drawn with a *p*-value < 0.05 which were performed by using GeoDa software (version 1.18).

#### Exploratory data analysis

Pearson correlation coefficients were calculated between population aging indicators and social-economic factors during the study period. The results showed that Pearson’s correlation coefficients were positively significant in most years based on two-tailed tests (see the supplementary tables).

## Results

### Spatial-temporal variations and distribution patterns

#### Overall spatial-temporal variations

**Fig. 1:**
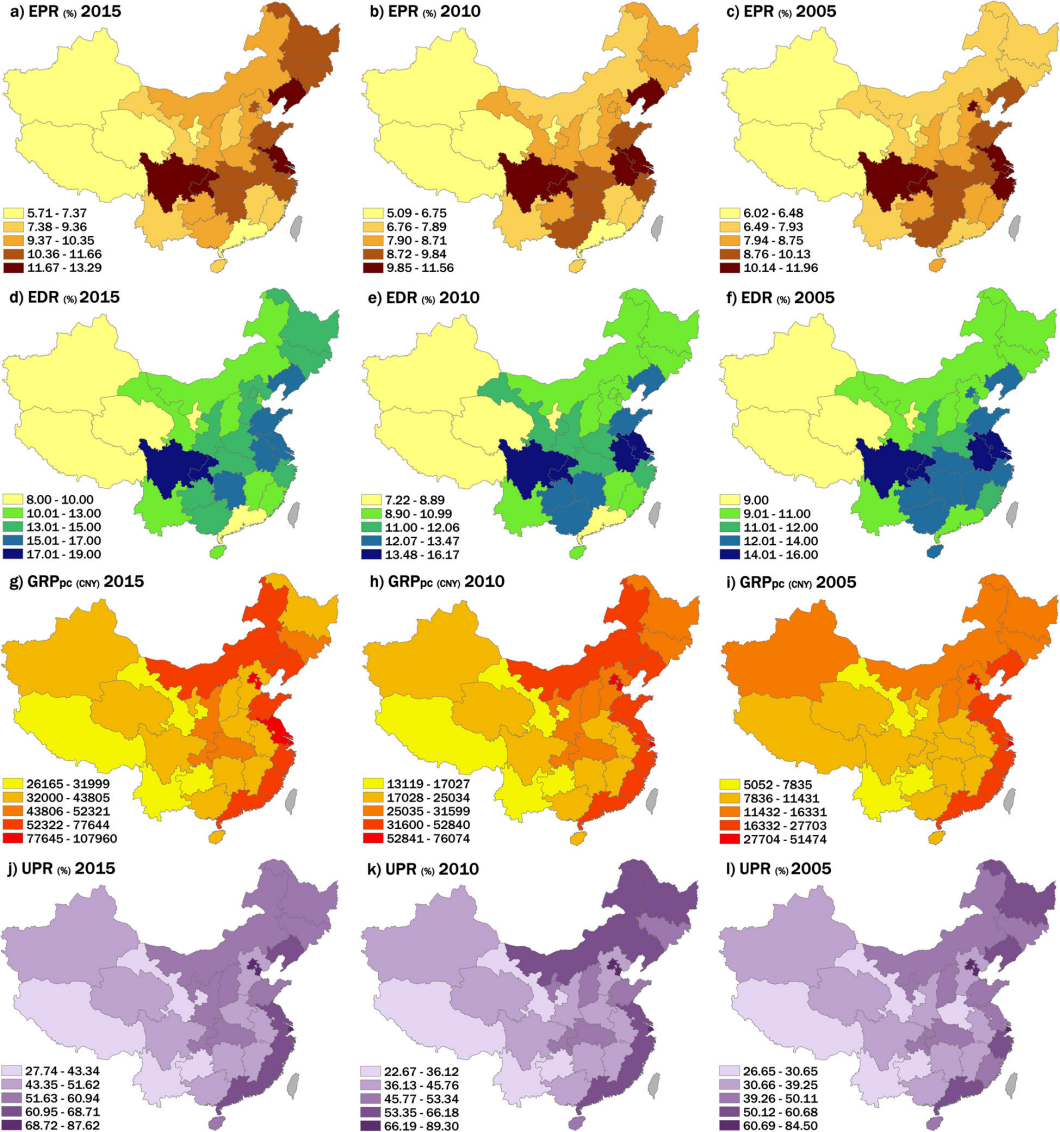
Spatial distribution and variation of EPR, EDR, GRP_pc_, and UPR at the provincial level in China in 2005, 2010, and 2015. The studied factors are divided into five levels based on natural breaks (Jenks) in ArcGIS. Data in Hong Kong, Macau, and Taiwan are not available in this study

The variation of population aging and social-economic factors at the provincial level in China with a five-year interval in 2005, 2010, and 2015 were depicted in Fig. 1. Several key points of the spatial-temporal variation trend can be concluded. First, the spatial difference of two population aging indicators showed a similarity that the eastern part of China (e.g., Liaoning, Beijing, Jiangsu) and Sichuan Province exhibited the highest values, and most provinces in the western part of China showed relatively lower values. Further, several provinces in southern China such as Guangdong and Fujian provinces showed relatively low values of EPR and EDR compared with the surrounding provinces (Fig. 1a-f). Second, the spatial difference of GRP_pc_ and UPR showed a similar trend that the high values most concentrated in the eastern coastal areas and northeastern part of China, while the low values were mainly in the western part of China (Fig. 1g-l). Additionally, the variation of these four indicators exhibited stable increasing trends in most provinces in China, and the spatial difference presented a stable trend that has not been changed substantially from 2002 to 2018.

#### Moran’s *I* statistics

**Fig. 2:**
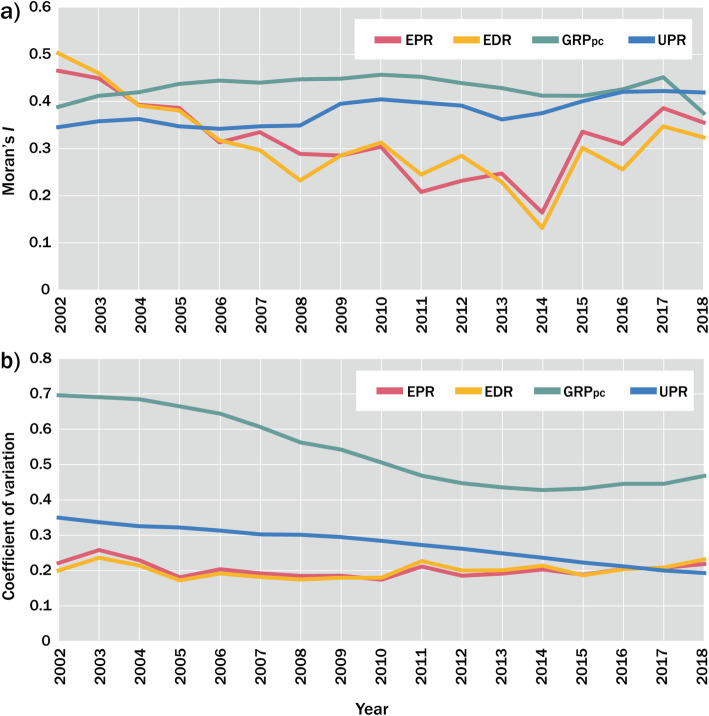
The variation of Moran’s *I* (a) and COV (b) of population aging and social-economic indicators in China from 2002 to 2018

To compare the global spatial autocorrelations of the four indicators, univariate Moran’s *I* statistics were calculated. The results showed that the Moran’s *I* indices of the four indicators were all significantly greater than zero, indicating that both population aging and social-economic indicators presented an obvious spatial agglomeration and significant positive spatial autocorrelation (i.e., cluster of provinces with similar indicators surrounded by provinces with similar values) at the provincial level in China from 2002 to 2018 (Table 2). Moreover, the annual variation of the Moran’s *I* indices of these indicators showed some difference (Fig. 2a). First, Moran’s *I* statistics of EPR and EDR showed a similar trend with continuous decreasing from the values around 0.5 to 0.1 from 2002 to 2014, and then these two indicators presented an increasing trend to 2018. Second, the GRP_pc_ and UPR exhibited a relatively stable trend with limited variation around the value of 0.4 (Fig. 2a).

**Table 2:** Univariate Moran’s *I* and coefficient of variation of population aging and social-economic indicators^a^

Year	Univariate Moran’s *I*				Coefficient of variation			
	EPR	EDR	GRP_pc_	UPR	EPR	EDR	GRP_pc_	UPR
2002	0.47	0.50	0.39	0.35	0.22	0.20	0.70	0.35
2003	0.45	0.46	0.41	0.36	0.26	0.24	0.69	0.34
2004	0.39	0.39	0.42	0.36	0.23	0.21	0.68	0.33
2005	0.39	0.38	0.44	0.35	0.18	0.17	0.66	0.32
2006	0.31	0.32	0.44	0.34	0.20	0.19	0.64	0.31
2007	0.33	0.30	0.44	0.35	0.19	0.18	0.61	0.30
2008	0.29	0.23	0.45	0.35	0.18	0.17	0.56	0.30
2009	0.29	0.29	0.45	0.40	0.19	0.18	0.54	0.29
2010	0.30	0.31	0.46	0.40	0.17	0.18	0.51	0.28
2011	0.21	0.24	0.45	0.40	0.21	0.23	0.47	0.27
2012	0.23	0.28	0.44	0.39	0.19	0.20	0.45	0.26
2013	0.25	0.23	0.43	0.36	0.19	0.20	0.44	0.25
2014	0.16	0.13	0.41	0.38	0.20	0.21	0.43	0.24
2015	0.34	0.30	0.41	0.40	0.19	0.19	0.43	0.22
2016	0.31	0.26	0.43	0.42	0.20	0.20	0.45	0.21
2017	0.39	0.35	0.45	0.42	0.21	0.21	0.45	0.20
2018	0.36	0.32	0.37	0.42	0.22	0.23	0.47	0.19

^a^All significant at the 95% confidence level

#### Coefficient of variation

COV values of the four indicators were calculated (Table 2) and depicted (Fig. 2b). Obvious differences between these indicators can be found. First, a stable trend of COV in EPR and EDR can be identified which was around the value of 0.2 (Fig. 2b). It indicated that the distribution and the dispersion degree of these two population-aging indicators did not change obviously during the study period. In comparison, COV of GRP_pc_ showed an obvious decline trend which was from 0.7 decreased to lower than 0.5, indicating the dispersion degree of GRP_pc_ declined during the study period. Furthermore, a slight decline trend can be found in UPR from higher than 0.3 to lower than 0.2 during the same period (Fig. 2b).

#### Kernel density estimation

**Fig. 3:**
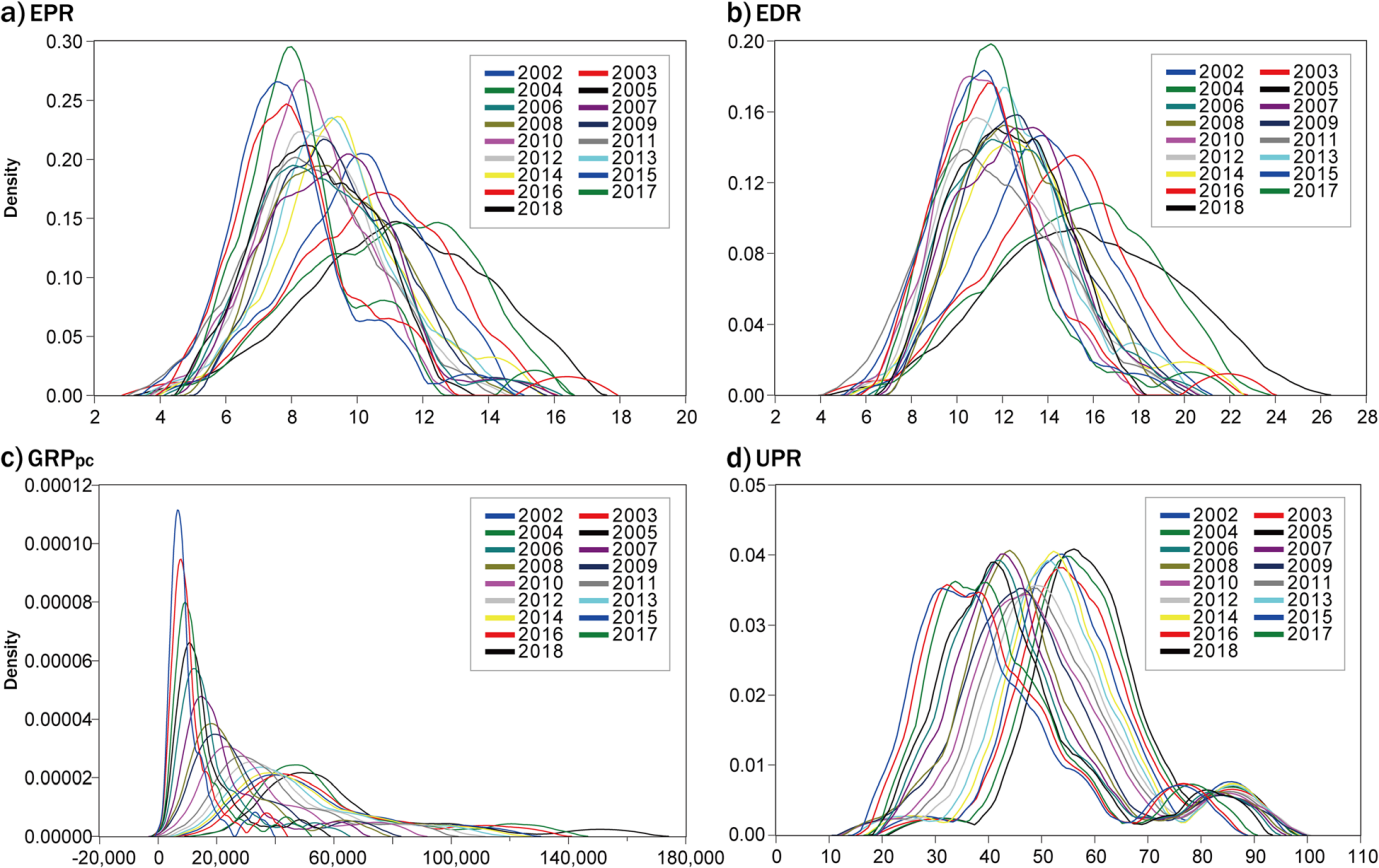
Kernel densities estimation curves of population aging indicators (a-b) and social-economic indicators (c-d) in China from 2002 to 2018

KDE distribution of the studied indicators was illustrated respectively (Fig. 3). The KDE curves of EPR and EDR presented similar patterns with bell-shaped curves, and the variation of the curves showed a slight decreasing trend of the peak values from 2002 to 2018, and the movement of the curves exhibited a right trend (Fig. 3 a, b). In addition, GRP_pc_ exhibited steep KDE curves with right-skewed distribution and long-tails, indicating stronger disparities among different provinces, and it showed a sharp decreasing trend of the peak values during the study period (Fig. 3c). Further, KDE curves of UPR showed a stable twin-peaked distribution and the curves moved to the right from 2002 to 2018 (Fig. 3d).

According to the analysis performed above, several features of the spatial-temporal variation and distribution patterns can be drawn. EPR and EDR showed a larger variety of spatial autocorrelation and stable trends of dispersion degree without an obvious convergence trend. In contrast, GRP_pc_ and UPR exhibited stable trends of spatial autocorrelation features. Specifically, GRP_pc_ showed the most obvious disparities and an obvious convergence trend, and UPR showed a slight convergence trend indexed by COV. Furthermore, convergence trends by COV are consistent with the KDE curves that EPR and EDR exhibited relatively small dispersion than GRP_pc_ which presented a repaid decline of the disparities over time.

### Identifying the movement of the gravity centers

**Fig. 4:**
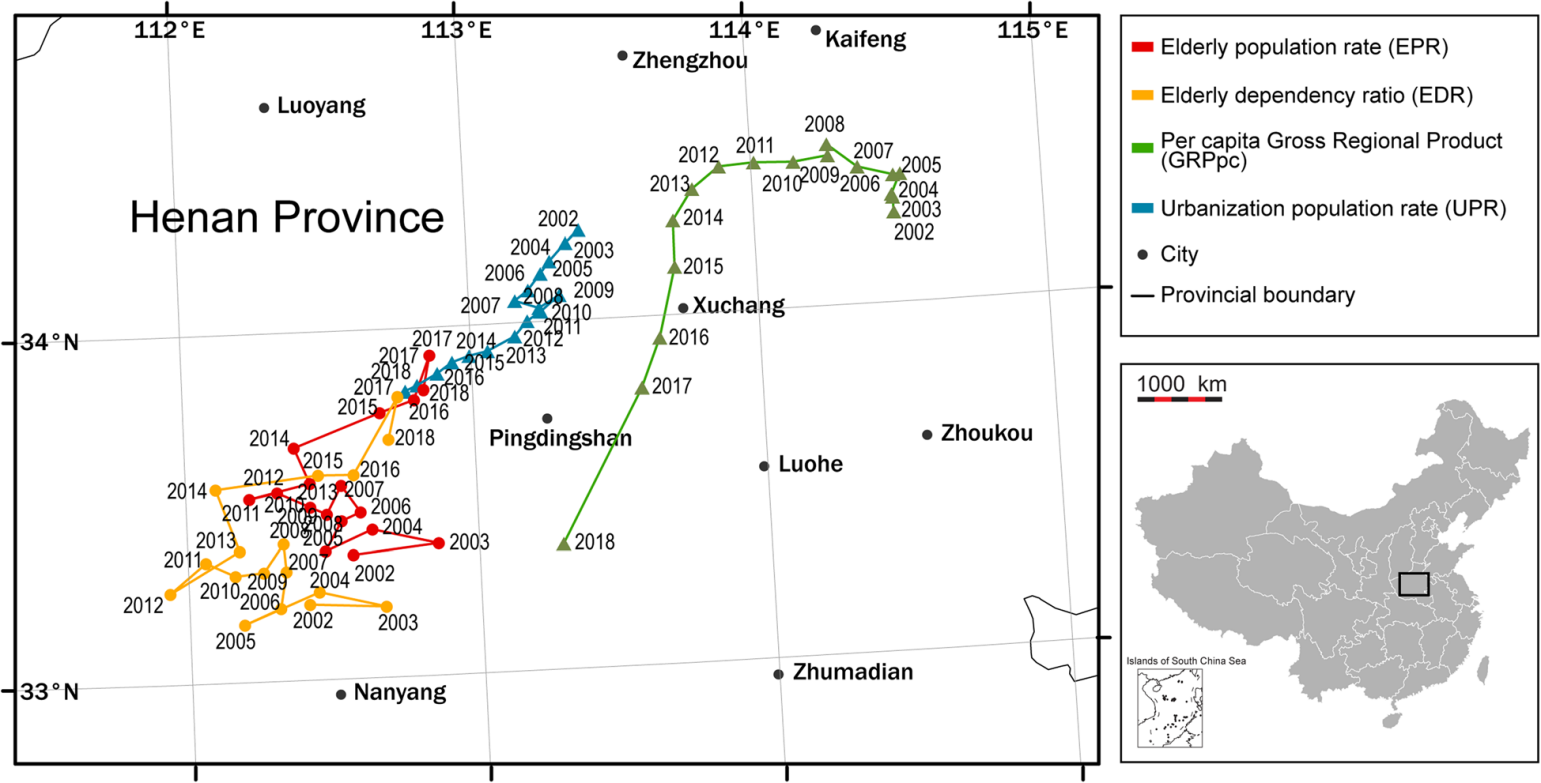
Annually movement of the gravity centers of population aging and social-economic indicators in China from 2002 to 2018

From 2002 to 2018, the gravity centers of EPR and EDR were mainly concentrated in the areas with latitude and longitude ranges between 112°E and 113°E and between 33°N and 34°N, which located in the middle of Henan Province in China (Fig. 4). Further, the movement of the gravity centers of EPR and EDR showed a similar pattern during the study period, which presented an overall movement towards the northwest and then to the northeast. The total movement distances of EPR and EDR were 226.87 and 281.30 km, with an average rate of 15.12 and 18.75 km/year, respectively. Meanwhile, the gravity centers of GRP_pc_ were mainly distributed to the northeast of the areas of EPR and EDR (Fig. 4). Additionally, the movement of the gravity centers of GRP_pc_ showed an opposite trend of the population aging indicators, which presented an overall movement towards the southwest. The total movement distance of GRP_pc_ was 206.81 km with an average rate of 13.79 km/year. In comparison, the gravity centers of UPR were mainly to the west of GRP_pc_, which concentrated in a limited area around 113°E and 34°N with a total movement distance of 103.37 km with an average rate of 6.89 km/year. The movement of the gravity centers of UPR exhibited a similar southwest toward with GRP_pc_ (Fig. 4).

Accordingly, several points can be obtained based on the gravity center analysis. First, the gravity centers of the four indicators were mainly concentrated in the middle of Henan Province, which is to the east of the geodetic origin of China (34°32′27″N, 108°55′25″E) located in Shaanxi Province. It can be pointed out that the gravity centers of these four indicators do not match the location of geometric centers at the national scale. Our findings further indicated that the population aging and social-economic factors were not evenly distributed in China. Second, both the gravity centers of the elderly population proportion and elderly dependency levels moved northeastward from 2002 to 2018, which showed similar directions and tracks. However, the economic and urbanization factors showed a southwestward movement, which exhibited obvious reverse evolution trends compared to the population aging indicators. Meanwhile, the distances between the gravity centers of population aging and social-economic factors became smaller during the study period. Furthermore, the movement rates of population aging indicators presented higher levels than that of GRP_pc_ and UPR annually. The movement rate of the economic factor showed a faster level with an accelerating trend especially in 2018.

### Spatial interaction between variables

**Fig. 5:**
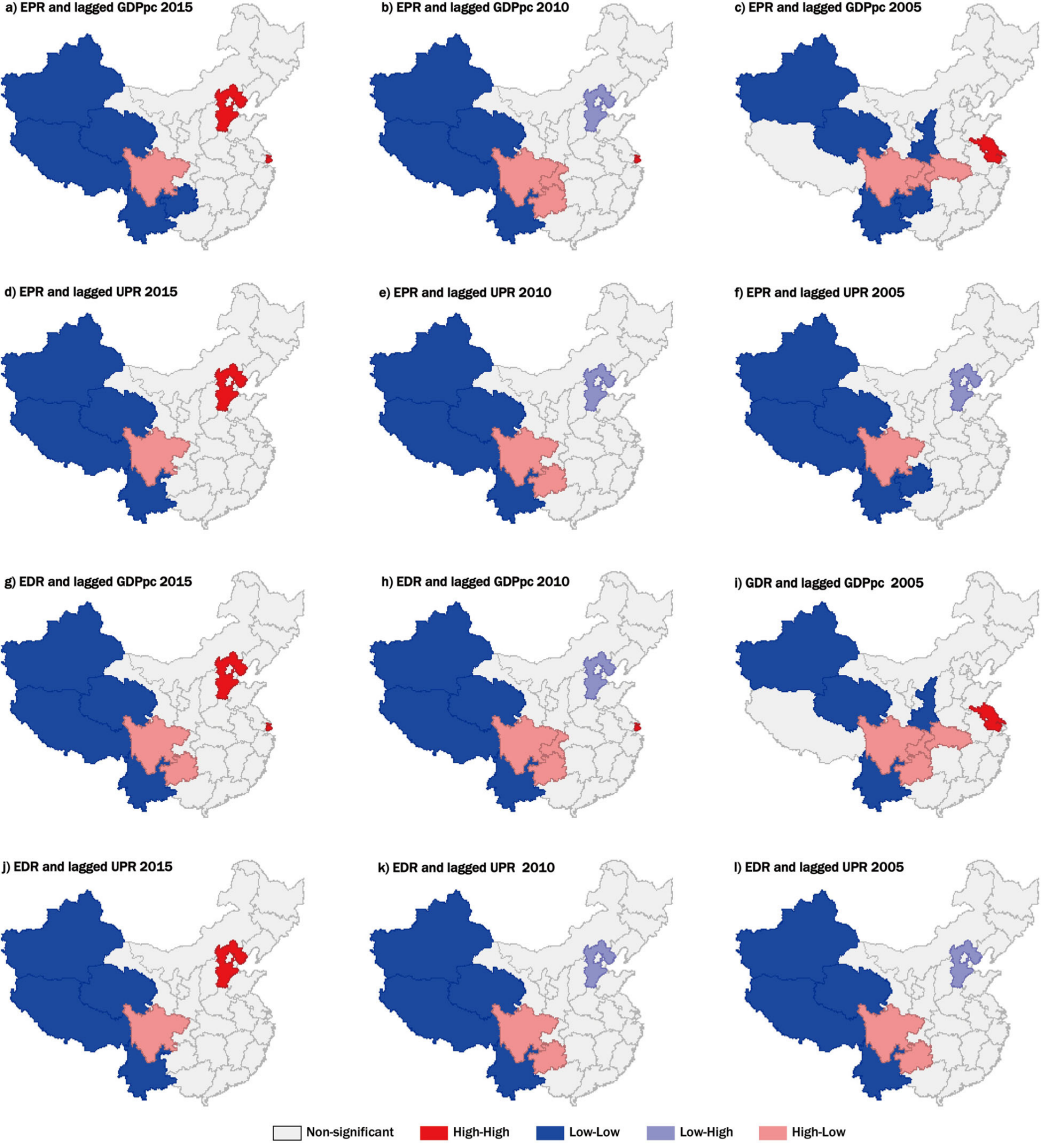
Spatial distribution and variation of bivariate Local Moran cluster (BiLISA) between population aging indicators and social-economic factors at the provincial level in China in 2005, 2010, and 2015. Islands in the dataset they would be shown as missing values because they have no adjacent neighbors

Spatial interaction was measured by bivariate Moran’s *I* between population aging indicators and social-economic factors (Table 3). The results demonstrated that the bivariate Moran’s *I* indices between the four indicators were all significantly greater than zero except for the period from 2010 to 2014. It indicated that the significant positive of the association between population aging variables and social-economic variables in neighboring regions. Further, BiLISA maps with a five-year interval in 2005, 2010, and 2015 were illustrated (Fig. 5). Based on the maps, the provinces with *p*-values below 0.05 were colored blue or red, and provinces with non-significant BiLISA statistics were colored gray (see map legend as well).

**Table 3:** Bivariate Moran’s *I* of population aging and social-economic indicators

	EPR-lagged GRP_pc_	EDR-lagged GRP_pc_	EPR-lagged UPR	EDR-lagged UPR
2018	0.22^a^	0.19^a^	0.26^a^	0.20^a^
2017	0.29^a^	0.25^a^	0.32^a^	0.26^a^
2016	0.29^a^	0.23^a^	0.30^a^	0.24^a^
2015	0.26^a^	0.18^a^	0.26^a^	0.18^a^
2014	0.09	−0.01	0.12	0.02
2013	0.16	0.05	0.18^a^	0.09
2012	0.08	−0.05	0.13	0.02
2011	0.07	−0.05	0.10	0.00
2010	0.17^a^	0.04	0.17^a^	0.06
2009	0.28^a^	0.21^a^	0.26^a^	0.19^a^
2008	0.32^a^	0.24^a^	0.29^a^	0.22^a^
2007	0.32^a^	0.24^a^	0.28^a^	0.20^a^
2006	0.31^a^	0.23^a^	0.25^a^	0.17^a^
2005	0.28^a^	0.17^a^	0.23^a^	0.12
2004	0.35^a^	0.30^a^	0.28^a^	0.23^a^
2003	0.37^a^	0.35^a^	0.29^a^	0.26^a^
2002	0.36^a^	0.33^a^	0.27^a^	0.23^a^

^a^Significant at the 95% confidence level

Several points of distribution patterns and variation trends can be obtained based on bivariate autocorrelation analysis. First, the significant Low-Low (LL) provinces were mainly located in the southwestern and northwestern parts of China, but Shaanxi and Guizhou Provinces were changed from LL areas to the non-significant area and Tibet was changed from non-significant to LL areas. In comparison, the High-High (HH) areas changed from Jiangsu Province in 2005 to Hebei Province in 2015 (Fig. 5). Second, it further suggested that in the provinces in western areas such as Xinjiang, Qinghai, Tibet, and Yunnan Provinces, the population aging variables in these provinces and the social-economic variables in the neighboring provinces are both at lower levels. In contrast, population aging variables in Jiangsu and Hebei Provinces and the social-economic variables in the neighboring provinces are at higher levels. Further, the change of the BiLISA in space indicated that the LL provinces showed a southwestern-direction movement and the HH provinces showed a northern movement, which is consistent with the opposite movement trends of gravity centers of the aging and social-economic factors in China.

## Discussion

The population aging level and social-economic development are closely related. In this study, four indicators were selected to represent the levels of population aging and social-economic development. Then, this study illustrated the marked spatial-temporal variability of population aging and social-economic indicators in China from 2002 to 2018. In addition, our findings identified significant positive spatial autocorrelation by Moran’s *I* as well as the obvious spatial disparities determined by kernel density estimation and coefficient of variation. Furthermore, spatial distribution and movement of gravity centers of the four indicators were investigated. Our study is one of the first that investigated the gravity center evolution of population aging and social-economic factors with spatial-temporal distribution patterns in China. In this section, we would like to further compare our results and discuss the future implications.

First, the spatial distribution pattern of population aging has been investigated by extensive studies, and a positive spatial autocorrelation was found at the global level [9] and the country level such as Japan [11], South Korea [12], Italy [49], and China [25]. Those papers indicated that the population showed wide-ranging multiplicity and place specificity with complex spatial-temporal processes [50], and the aging population between regions can be attributed to their proximity to one another [9]. The results in this study are mainly consistent with the results from the previous studies, which demonstrated that population aging indicators including EPR and EDR cluster in space at the national level from 2002 to 2018. Moreover, the temporal variation of spatial autocorrelation indexed by Moran’s *I* and distribution patterns indexed by COV and KDE of the population aging and social-economic factors during the study period was illustrated in this paper. For the first time, our results demonstrated the different variation features among these factors which showed opposite variation trends between the population aging and social-economic factors in China. Specifically, this study revealed a larger variety of spatial autocorrelation and stable trends of dispersion degree without convergence in EPR and EDR. In contrast, spatial autocorrelation of GRP_pc_ and UPR exhibited stable trends and dispersion degree showed obvious convergence during the study period from 2002 to 2018 in China. The analysis conducted above demonstrated the difference in the spatial-temporal distribution and variation patterns between population aging and social-economic factors.

Furthermore, our study revealed an opposite trend of the gravity centers movement in space of the aging and social-economic factors in China. According to previous studies, the economic gravity centers moved from the north to the south in China, and the movement of total population gravity centers showed a southwest forward, indicating a similar movement trend of the economy and population [51, 52]. The results of the current study found that the gravity centers of the elderly population proportion and elderly dependency levels moved northeastward from 2002 to 2018, which was opposite of the movement direction of social-economic factors, though the distances between the gravity centers of population aging and social-economic factors became smaller. Moreover, the average movement rates of population aging indicators were higher than that of GRP_pc_ and UPR during the study period. The difference of the gravity center movement directions and movement rates further indicates the imbalance patterns may extend and the social-economic burdens of population aging may become increased in China in the future.

Additionally, spatial interaction between the variables can provide more information. Our results detected significant positive associations between population aging variables and social-economic variables in neighboring regions by using bivariate Moran’s *I.* It may indicate that the population aging variables in a province have a spillover effect on the social-economic variables in its neighboring provinces. Further, the spatial visualization of the BiLISA and its variation across time is in line with the movement trends of gravity centers of the aging and social-economic factors in China. Thus, the spatial interaction between variables during the study period demonstrated a polarization trend that the difference between southwest and northeast directions of China exhibited a tendency to expand from 2002 to 2018 and it may be widened over time.

More broadly, our results further demonstrated several considerations for policymakers. Firstly, the regional unbalance of population aging and social-economic development in China has been reflected by the spatial-temporal variation and distribution patterns. It was suggested that policymakers should consider the consequences of economic policies for population aging [53]. Hence, a coordinated development plan should be considered by the central and local governments in China. Secondly, the regional unbalance trend of the population aging and social-economic levels was detected by the movement of gravity center analysis. Importantly, both the gravity centers of the elderly population proportion and elderly dependency levels moved northeastward, which were opposite to the total population and social-economic development in China which showed southwestward movement directions of gravity centers [51, 52]. Further, these opposite patterns exhibited continuous trends, which may result in the increase of the financial burden of pension in northern China in the future. Hence, the government should focus on the spatial disparity of social-economic development levels and reasonable distribution of old-aged supporting resources across regions especially in northern China.

There are some limitations to this study. First, our empirical results are based on the provincial-level data, and a local scale (e.g., prefecture and county levels) was not performed in this study, which limited our understanding of the spatial-temporal distribution of population aging and social-economic indicators. Hence, future studies on the finer spatial scale will be meaningful to reveal the refined patterns of population aging levels in China. Second, although we captured the temporal evolution of the gravity centers movement of the four indicators, a more extensive study such as the coordination degree analysis of population aging and social-economic development with spatial-temporal modeling would be extremely valuable. Third, natural environmental factors (e.g., heat and air pollution) also showed a strong influence on the elderly [54, 55], which indicates that we cannot ignore the indirect effects of the natural environment factors on population aging. Moreover, the Chinese population is experiencing a rapid aging process, thus, it will be interesting to compare our results in this paper to other countries at different stages of aging and social-economic development levels.

## Conclusions

This study illustrated the spatial-temporal distribution of population aging indicators (elderly population rate and elderly dependency ratio) and social-economic factors (per capita Gross Regional Product and urbanization population rate) and explored the spatial distribution and movement of gravity centers of the four indicators across the provinces in China from 2002 to 2018. Several major conclusions were drawn as follows:

1. It was revealed a larger variety of global spatial autocorrelation indexed by Moran’s *I* and stable trends of dispersion degree without obvious convergence in EPR and EDR. In contrast, GRP_pc_ and UPR exhibited stable trends of spatial autocorrelation features. Specifically, GRP_pc_ showed the most obvious disparities and an obvious convergence trend, and UPR showed a slight convergence trend indexed by COV. Furthermore, convergence trends by COV were consistent with the KDE curves that EPR and EDR exhibited relatively small dispersion degrees than GRP_pc_ which presented a repaid decline of the disparities over time.
2. It further indicated that the population aging and social-economic factors are not evenly distributed in China. Specifically, both the proportion of the elderly population and elderly dependency levels centers of gravity moved northeastward from 2002 to 2018, which indicated a similar direction of the two indicators. However, the economic and urbanization factors showed a southwestward movement, which exhibited obvious reverse evolution trends than the population aging indicators. The distances between the gravity centers of population aging and social-economic factors became smaller during the study period. Furthermore, the annual movement rates of population aging indicators are higher than GRP_pc_ and UPR. The movement rate of the economic factor showed a faster level with an accelerating trend especially in 2018.
3. Our findings revealed the difference in spatio-temporal distribution and variation between population aging and social-economic factors. Further, the spatial visualization of the BiLISA and its variation is in line with the movement trends of gravity centers which showed a polarization trend that the difference between southwest and northeast directions of China exhibited a tendency to expand from 2002 to 2018 and it may be widened over time. Thus, it may exacerbate the social-economic burden of elderly care in northern China in the coming intensified aging society. Hence, future development policy should focus on the economic growth and the reasonable distribution of old-aged supporting resources across the regions especially in northern China.

## Supplementary Information

**Additional file 1: Supplementary Table 1**. Pearson correlation coefficients between EPR and social-economic factors (GRP_pc_ and UR) at provincial-level of China (two tailed test). **Supplementary Table 2**. Pearson correlation coefficients between EDR and social-economic factors (GRP_pc_ and UR) at provincial-level of China (two tailed test).

## Data Availability

All the data used in this study are from the national databases of China which are publicly available, and unrestricted re-use is permitted. The demographic data in 2010 are from the Tabulation on the 2010 Population Census of the People’s Republic of China released by the Population Census Office under the Stata Council of China and Department of Population and Employment Statistics under the National Bureau of Statistics of China (http://www.stats.gov.cn/tjsj/pcsj/rkpc/6rp/indexch.htm). The sample survey data of 1% and 1‰ population and social-economic data are from the database of China Statistical Yearbooks released by the National Bureau of Statistics of China (http://www.stats.gov.cn/english/Statisticaldata/AnnualData/).

## Abbreviations

BiLISA: Bivariate local indicators of spatial association
COV: Coefficient of variation
EDR: Elderly dependency ratio
EPR: Elderly population rate
GRP_pc_: Per capita Gross Regional Product
KDE: Kernel density estimation
UPR: Urban population rate

## References

[1] United Nations (2017). World population prospects 2017.

[2] Lindh T, Malmberg B (1999). Age structure effects and growth in the OECD, 1950–1990. J Popul Econ.

[3] Bloom DE, Canning D, Fink G (2010). Implications of population ageing for economic growth. Oxf Rev Econ Policy.

[4] Harper S (2014). Economic and social implications of aging societies. Science..

[5] Sheiner L (2014). The determinants of the macroeconomic implications of aging. Am Econ Rev.

[6] Bloom DE, Chatterji S, Kowal P, Lloyd-Sherlock P, McKee M, Rechel B, Rosenberg L, Smith JP (2015). Macroeconomic implications of population ageing and selected policy responses. Lancet.

[7] United Nations Population Fund (2012). Ageing in the Twenty-First Century: A Celebration and A Challenge.

[8] Li J, Han X, Zhang X, Wang S (2019). Spatiotemporal evolution of global population ageing from 1960 to 2017. BMC Public Health.

[9] Wang S (2020). Spatial patterns and social-economic influential factors of population aging: a global assessment from 1990 to 2010. Soc Sci Med.

[10] Yu T (2013). China’s aging population and its spatial features in city areas (2000–2010).

[11] Shiode N, Morita M, Shiode S, Okunuki K (2014). Urban and rural geographies of aging: a local spatial correlation analysis of aging population measures. Urban Geogr.

[12] Yeo C-H, Seo Y-H (2014). An analysis on the spatial spillover patterns of aging population in rural areas. J Korean Assoc Geogr Inf Stud.

[13] Diaconu L (2015). Ageing population: comparative analysis among European Union states. CES Working Papers.

[14] Kumler MP, Goodchild MF (1992). The population center of Canada-just north of Toronto.

[15] Hilgard JE (1872). The advance of population in the United States. Scribners Monthly.

[16] Sviatlovsky EE, Eells WC (1937). The centrographical method and regional analysis. Geogr Rev.

[17] Jones BG (1980). Applications of centrographic techniques to the study of urban phenomena: Atlanta, Georgia 1940–1975. Econ Geogr.

[18] United Nations (2015). World population ageing 2015.

[19] United Nations (2019). World population ageing 2019.

[20] Zhang K, Chen N (2014). Characteristics of spatial-temporal evolution in population aging and driving mechanism at county level in Fujian Province during 1990-2010. Prog Geogr.

[21] Li S, Cheng Y, Gao SY (2017). The Regional Difference of Population Aging in Beijing-Tianjin-Hebei Region.

[22] Han X, Li J, Wang N (2018). Spatiotemporal evolution of Chinese ageing from 1992 to 2015 based on an improved Bayesian space-time model. BMC Public Health.

[23] Xu X, Zhao Y, Zhang X, Xia S (2018). Identifying the impacts of social, economic, and environmental factors on population aging in the Yangtze River Delta using the geographical detector technique. Sustainability..

[24] Cheng Y, Gao S, Li S, Zhang Y, Rosenberg M (2019). Understanding the spatial disparities and vulnerability of population aging in China. Asia Pac Policy Stud.

[25] Wu Y, Song Y, Yu T (2019). Spatial differences in China’s population aging and influencing factors: the perspectives of spatial dependence and spatial heterogeneity. Sustainability..

[26] National Bureau of Statistics of China (2010). Tabulation on the 2010 population census.

[27] Roberts AW, Ogunwole SU, Blakeslee L, Rabe MA. The population 65 years and older in the United States. Am Community Surv Rep. 2016.

[28] Harper S, Laws G (1995). Rethinking the geography of ageing. Prog Hum Geogr.

[29] OECD (2019). Elderly population (indicator).

[30] McNicoll G (2002). World population ageing 1950-2050. Popul Dev Rev.

[31] Hui ECM, Zheng X, Hu J (2012). Housing price, elderly dependency and fertility behaviour. Habitat Int.

[32] Han X, Cheng Y (2020). Consumption- and productivity-adjusted dependency ratio with household structure heterogeneity in China. J Econ Ageing.

[33] Centers for Disease Control and Prevention (2003). Trends in aging--United States and worldwide. MMWR Morb Mortal Wkly Rep.

[34] Bloom DE, Eggleston KN. The economic implications of population ageing in China and India: Introduction to the special issue. J Econ Ageing. 2014:1–7.

[35] Anselin L (1995). Local indicators of spatial association—LISA. Geogr Anal.

[36] Griffith DA (2013). Spatial autocorrelation and spatial filtering: gaining understanding through theory and scientific visualization.

[37] Yang G, Li W, Wang J, Zhang D (2016). A comparative study on the influential factors of China’s provincial energy intensity. Energy Policy.

[38] Gruebner O, Khan M, Burkart K, Lautenbach S, Lakes T, Krämer A, Subramanian SV, Galea S (2017). Spatial variations and determinants of infant and under-five mortality in Bangladesh. Health Place.

[39] Zhang Y, Fu Y, Kong X, Zhang F (2019). Prefecture-level city shrinkage on the regional dimension in China: spatiotemporal change and internal relations. Sustain Cities Soc.

[40] Cliff AD, Ord JK (1981). Spatial processes: models & applications.

[41] Silverman BW (2018). Density estimation for statistics and data analysis.

[42] Adhikari D, Chen Y (2014). Energy productivity convergence in Asian countries: a spatial panel data approach. Int J Econ Financ.

[43] Goschin Z (2014). Regional inequalities and sigma divergence in Romania. Procedia Econ Finance.

[44] Searls DT (1964). The utilization of a known coefficient of variation in the estimation procedure. J Am Stat Assoc.

[45] Zhang Y, Zhang J, Yang Z, Li J (2012). Analysis of the distribution and evolution of energy supply and demand centers of gravity in China. Energy Policy.

[46] Chen J, Xu C, Li K, Song M (2018). A gravity model and exploratory spatial data analysis of prefecture-scale pollutant and CO2 emissions in China. Ecol Indic.

[47] Chohan UW (2018). The political economy of OBOR and the global economic Center of Gravity.

[48] Anselin L, Syabri I, Smirnov O (2002). Visualizing multivariate spatial correlation with dynamically linked windows.

[49] Reynaud C, Miccoli S, Lagona F (2018). Population ageing in Italy: an empirical analysis of change in the ageing index across space and time. Spatial Demography.

[50] Rishworth A, Elliott SJ (2019). Global environmental change in an aging world: the role of space, place and scale. Soc Sci Med.

[51] Xu Y, Li S (2005). Dynamic evolvement of the population and the social economy gravity center in China. Hum Geogr.

[52] Lian X (2007). Analysis on the space evolvement track of population gravity center, employment gravity center and economic gravity center. Population J.

[53] Lloyd-Sherlock P (2000). Population ageing in developed and developing regions: implications for health policy. Soc Sci Med.

[54] Rupa B (2002). Samet Jonathan M. an exposure assessment study of ambient heat exposure in an elderly population in Baltimore, Maryland. Environ Health Perspect.

[55] Huang J, Li J, Yin P, Wang L, Pan X, Zhou M, Li G (2021). Ambient nitrogen dioxide and years of life lost from chronic obstructive pulmonary disease in the elderly: a multicity study in China. Chemosphere..

