# Supplementary material for: Exploring the spatial-temporal distribution and evolution of population aging and social-economic indicators in China
Source: BMC Public Health. 2021 May 22;21:966. doi: 10.1186/s12889-021-11032-z (PMC8140474; doi:10.1186/s12889-021-11032-z)
Supplement: Supplementary file 1 — Additional file 1: Supplementary Table 1. Pearson correlation coefficients between EPR and social-economic factors (GRPpc and UR) at provincial-level of China (two tailed test). Supplementary Table 2. Pearson correlation coefficients between EDR and social-economic factors (GRPpc and UR) at provincial-level of China (two tailed test). [file 12889_2021_11032_MOESM1_ESM.docx]

## Supplementary Table 1

Pearson correlation coefficients between EPR and social-economic factors (GDPpc and UR) at provincial-level of China (two tailed test).

|  | EPR2018 | EPR2017 | EPR2016 | EPR2015 | EPR2014 | EPR2013 | EPR2012 | EPR2011 | EPR2010 | EPR2009 | EPR2008 | EPR2007 | EPR2006 | EPR2005 | EPR2004 | EPR2003 | EPR2002 |
| --- | --- | --- | --- | --- | --- | --- | --- | --- | --- | --- | --- | --- | --- | --- | --- | --- | --- |
| GDPpc2018 | 0.27 |  |  |  |  |  |  |  |  |  |  |  |  |  |  |  |  |
| GDPpc2017 |  | 0.41** |  |  |  |  |  |  |  |  |  |  |  |  |  |  |  |
| GDPpc2016 |  |  | 0.40** |  |  |  |  |  |  |  |  |  |  |  |  |  |  |
| GDPpc2015 |  |  |  | 0.39* |  |  |  |  |  |  |  |  |  |  |  |  |  |
| GDPpc2014 |  |  |  |  | 0.24 |  |  |  |  |  |  |  |  |  |  |  |  |
| GDPpc2013 |  |  |  |  |  | 0.29 |  |  |  |  |  |  |  |  |  |  |  |
| GDPpc2012 |  |  |  |  |  |  | 0.17 |  |  |  |  |  |  |  |  |  |  |
| GDPpc2011 |  |  |  |  |  |  |  | 0.11 |  |  |  |  |  |  |  |  |  |
| GDPpc2010 |  |  |  |  |  |  |  |  | 0.25 |  |  |  |  |  |  |  |  |
| GDPpc2009 |  |  |  |  |  |  |  |  |  | 0.53** |  |  |  |  |  |  |  |
| GDPpc2008 |  |  |  |  |  |  |  |  |  |  | 0.54** |  |  |  |  |  |  |
| GDPpc2007 |  |  |  |  |  |  |  |  |  |  |  | 0.56** |  |  |  |  |  |
| GDPpc2006 |  |  |  |  |  |  |  |  |  |  |  |  | 0.61** |  |  |  |  |
| GDPpc2005 |  |  |  |  |  |  |  |  |  |  |  |  |  | 0.53** |  |  |  |
| GDPpc2004 |  |  |  |  |  |  |  |  |  |  |  |  |  |  | 0.77** |  |  |
| GDPpc2003 |  |  |  |  |  |  |  |  |  |  |  |  |  |  |  | 0.81** |  |
| GDPpc2002 |  |  |  |  |  |  |  |  |  |  |  |  |  |  |  |  | 0.77** |
| UR2018 | 0.41** |  |  |  |  |  |  |  |  |  |  |  |  |  |  |  |  |
| UR2017 |  | 0.51** |  |  |  |  |  |  |  |  |  |  |  |  |  |  |  |
| UR2016 |  |  | 0.51** |  |  |  |  |  |  |  |  |  |  |  |  |  |  |
| UR2015 |  |  |  | 0.47** |  |  |  |  |  |  |  |  |  |  |  |  |  |
| UR2014 |  |  |  |  | 0.31* |  |  |  |  |  |  |  |  |  |  |  |  |
| UR2013 |  |  |  |  |  | 0.38* |  |  |  |  |  |  |  |  |  |  |  |
| UR2012 |  |  |  |  |  |  | 0.22 |  |  |  |  |  |  |  |  |  |  |
| UR2011 |  |  |  |  |  |  |  | 0.15 |  |  |  |  |  |  |  |  |  |
| UR2010 |  |  |  |  |  |  |  |  | 0.30* |  |  |  |  |  |  |  |  |
| UR2009 |  |  |  |  |  |  |  |  |  | 0.53** |  |  |  |  |  |  |  |
| UR2008 |  |  |  |  |  |  |  |  |  |  | 0.61** |  |  |  |  |  |  |
| UR2007 |  |  |  |  |  |  |  |  |  |  |  | 0.59** |  |  |  |  |  |
| UR2006 |  |  |  |  |  |  |  |  |  |  |  |  | 0.60** |  |  |  |  |
| UR2005 |  |  |  |  |  |  |  |  |  |  |  |  |  | 0.50** |  |  |  |
| UR2004 |  |  |  |  |  |  |  |  |  |  |  |  |  |  | 0.65** |  |  |
| UR2003 |  |  |  |  |  |  |  |  |  |  |  |  |  |  |  | 0.69** |  |
| UR2002 |  |  |  |  |  |  |  |  |  |  |  |  |  |  |  |  | 0.65** |

* *P* < 0.05. ** *P* < 0.01.

## Supplementary Table 2

Pearson correlation coefficients between EDR and social-economic factors (GDPpc and UR) at provincial-level of China (two tailed test).

|  | EDR2018 | EDR2017 | EDR2016 | EDR2015 | EDR2014 | EDR2013 | EDR2012 | EDR2011 | EDR2010 | EDR2009 | EDR2008 | EDR2007 | EDR2006 | EDR2005 | EDR2004 | EDR2003 | EDR2002 |
| --- | --- | --- | --- | --- | --- | --- | --- | --- | --- | --- | --- | --- | --- | --- | --- | --- | --- |
| GDPpc2018 | 0.17 |  |  |  |  |  |  |  |  |  |  |  |  |  |  |  |  |
| GDPpc2017 |  | 0.30* |  |  |  |  |  |  |  |  |  |  |  |  |  |  |  |
| GDPpc2016 |  |  | 0.28 |  |  |  |  |  |  |  |  |  |  |  |  |  |  |
| GDPpc2015 |  |  |  | 0.22 |  |  |  |  |  |  |  |  |  |  |  |  |  |
| GDPpc2014 |  |  |  |  | 0.07 |  |  |  |  |  |  |  |  |  |  |  |  |
| GDPpc2013 |  |  |  |  |  | 0.09 |  |  |  |  |  |  |  |  |  |  |  |
| GDPpc2012 |  |  |  |  |  |  | -0.07 |  |  |  |  |  |  |  |  |  |  |
| GDPpc2011 |  |  |  |  |  |  |  | -0.09 |  |  |  |  |  |  |  |  |  |
| GDPpc2010 |  |  |  |  |  |  |  |  | 0.01 |  |  |  |  |  |  |  |  |
| GDPpc2009 |  |  |  |  |  |  |  |  |  | 0.38* |  |  |  |  |  |  |  |
| GDPpc2008 |  |  |  |  |  |  |  |  |  |  | 0.33* |  |  |  |  |  |  |
| GDPpc2007 |  |  |  |  |  |  |  |  |  |  |  | 0.38* |  |  |  |  |  |
| GDPpc2006 |  |  |  |  |  |  |  |  |  |  |  |  | 0.45** |  |  |  |  |
| GDPpc2005 |  |  |  |  |  |  |  |  |  |  |  |  |  | 0.26* |  |  |  |
| GDPpc2004 |  |  |  |  |  |  |  |  |  |  |  |  |  |  | 0.68** |  |  |
| GDPpc2003 |  |  |  |  |  |  |  |  |  |  |  |  |  |  |  | 0.75** |  |
| GDPpc2002 |  |  |  |  |  |  |  |  |  |  |  |  |  |  |  |  | 0.70** |
| UR2018 | 0.27 |  |  |  |  |  |  |  |  |  |  |  |  |  |  |  |  |
| UR2017 |  | 0.39* |  |  |  |  |  |  |  |  |  |  |  |  |  |  |  |
| UR2016 |  |  | 0.37* |  |  |  |  |  |  |  |  |  |  |  |  |  |  |
| UR2015 |  |  |  | 0.31* |  |  |  |  |  |  |  |  |  |  |  |  |  |
| UR2014 |  |  |  |  | 0.13 |  |  |  |  |  |  |  |  |  |  |  |  |
| UR2013 |  |  |  |  |  | 0.18 |  |  |  |  |  |  |  |  |  |  |  |
| UR2012 |  |  |  |  |  |  | -0.03 |  |  |  |  |  |  |  |  |  |  |
| UR2011 |  |  |  |  |  |  |  | -0.07 |  |  |  |  |  |  |  |  |  |
| UR2010 |  |  |  |  |  |  |  |  | 0.05 |  |  |  |  |  |  |  |  |
| UR2009 |  |  |  |  |  |  |  |  |  | 0.38* |  |  |  |  |  |  |  |
| UR2008 |  |  |  |  |  |  |  |  |  |  | 0.42** |  |  |  |  |  |  |
| UR2007 |  |  |  |  |  |  |  |  |  |  |  | 0.43** |  |  |  |  |  |
| UR2006 |  |  |  |  |  |  |  |  |  |  |  |  | 0.45** |  |  |  |  |
| UR2005 |  |  |  |  |  |  |  |  |  |  |  |  |  | 0.25 |  |  |  |
| UR2004 |  |  |  |  |  |  |  |  |  |  |  |  |  |  | 0.54** |  |  |
| UR2003 |  |  |  |  |  |  |  |  |  |  |  |  |  |  |  | 0.62** |  |
| UR2002 |  |  |  |  |  |  |  |  |  |  |  |  |  |  |  |  | 0.56** |

* *P* < 0.05. ** *P* < 0.01.
